# Supplementary material for: The BCAT1 CXXC Motif Provides Protection against ROS in Acute Myeloid Leukaemia Cells
Source: Antioxidants (Basel). 2022 Mar 31;11(4):683. doi: 10.3390/antiox11040683 (PMC9030579; doi:10.3390/antiox11040683)
Supplement: Supplementary file 1 [file antioxidants-11-00683-s001.zip › antioxidants-1624686-supplementary.pdf]

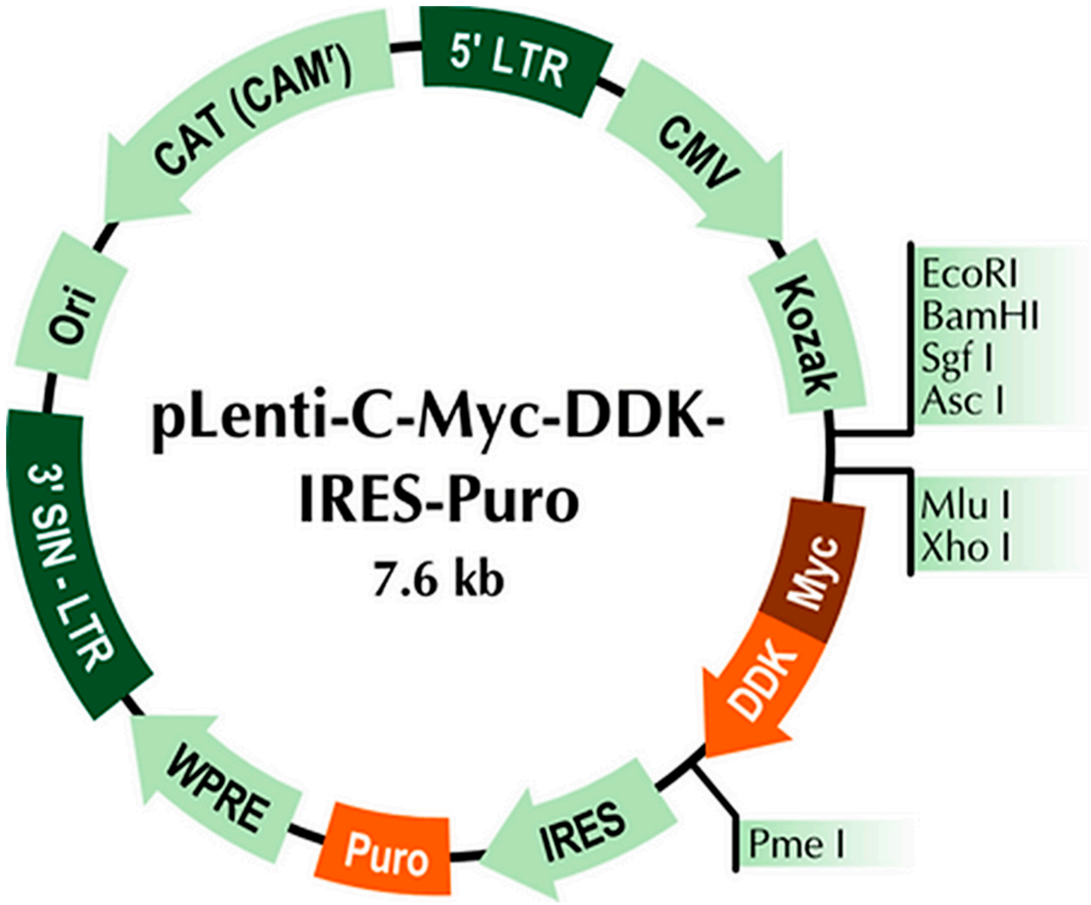

Supplemental Figure S1. OriGene pLenti-C-Myc-DDK-IRES-Puro vector map. WT and CXXC motif mutant *BCAT1* gene were cloned into the *Sgf*I and *Mlu*I restriction site.

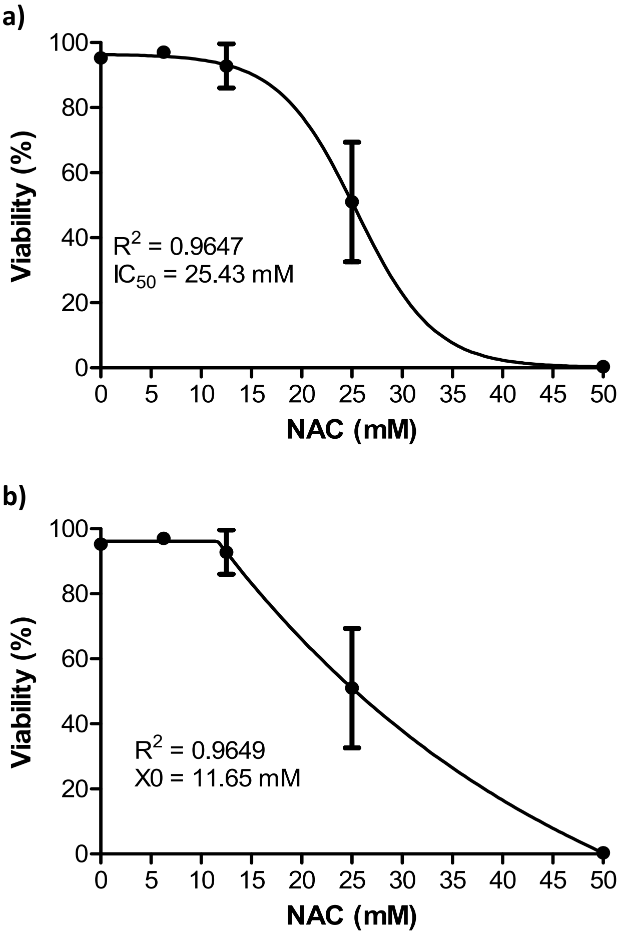

Supplemental Figure S2. N-acetyl cysteine (NAC) does response. a) Chart illustrates the IC_{50} for NAC using vector control U937 cells. **b)** Illustrating the minimum inhibitor concentration (X0) for NAC using vector control U937 cells. Dose responses were carried out in 10% FCS. Data presented are mean ± SD and analysed by non-linear regression (n=3).

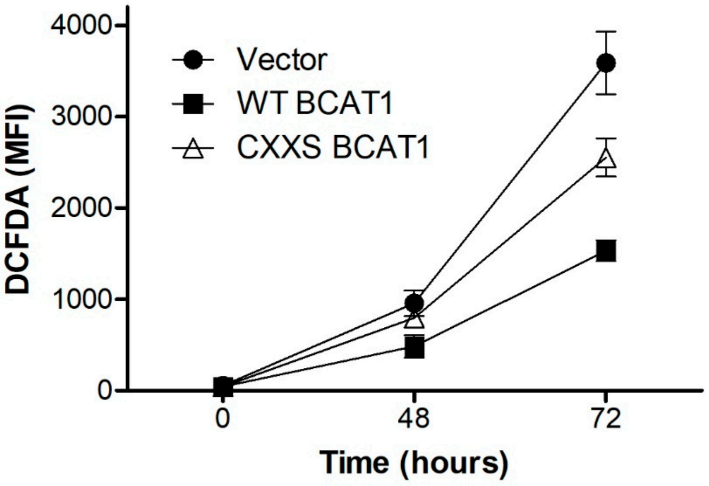

Supplemental Figure S3. The effect of serum starvation on cellular ROS as measured by DCFDA. Chart illustrates the difference in DCFDA signal between vector control, WT BCAT1 and CXXS BCAT1 overexpressing U937 cells at various time points. Data are mean ± SD (n=3).

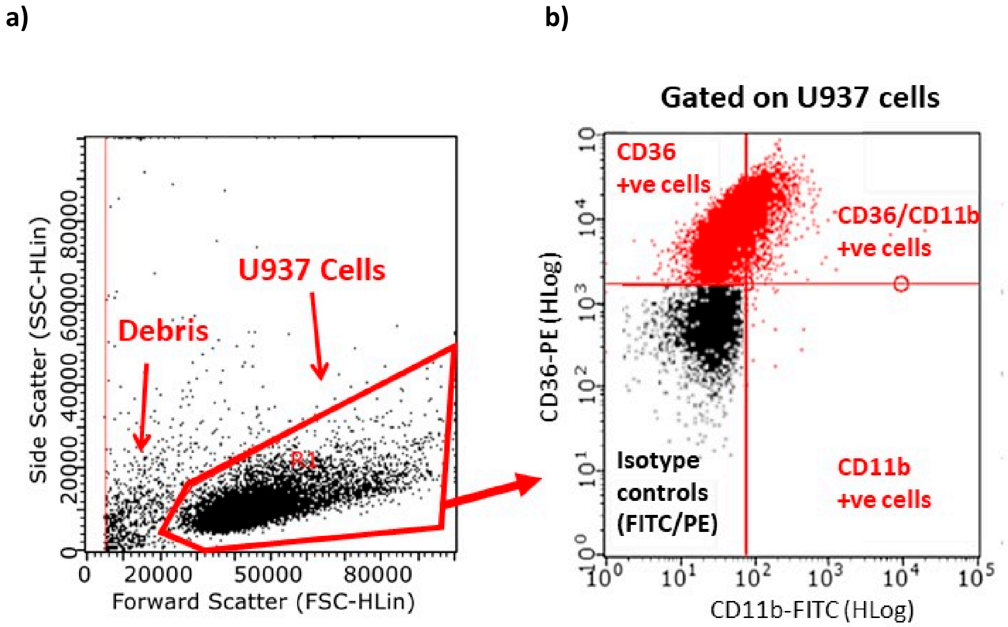

Supplemental Figure S4. Representative U937 cell flow cytometric gating strategy for CD11b/CD36 bivariate analysis. a) U937 cells (vector controls, WT BCAT1 and CXXS BCAT1) were gated based on forward and side scatter as illustrated, avoiding debris. **b)** The negative CD11b-FITC and CD36-PE quadrant was set based on the respective immunoglobulin/fluorophore isotypic control. CD36 +ve (positive) cells appear in the upper left quadrant, CD11b +ve cells appear in the lower right quadrant and CD36/CD11b double +ve cells appear in the upper right quadrant. The characteristic data shape indicates that CD36 expression increases CD11b expression following PMA treatment.

a)

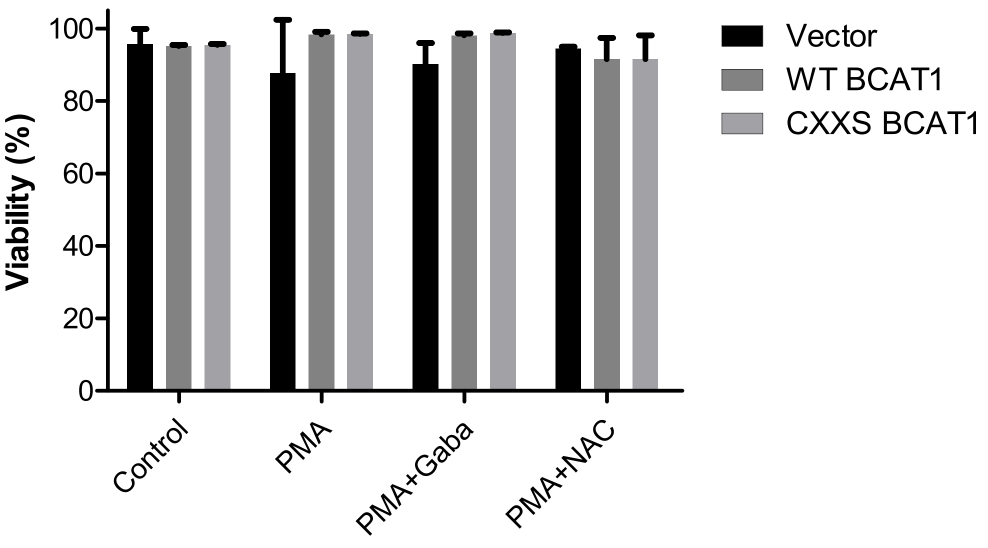

b)

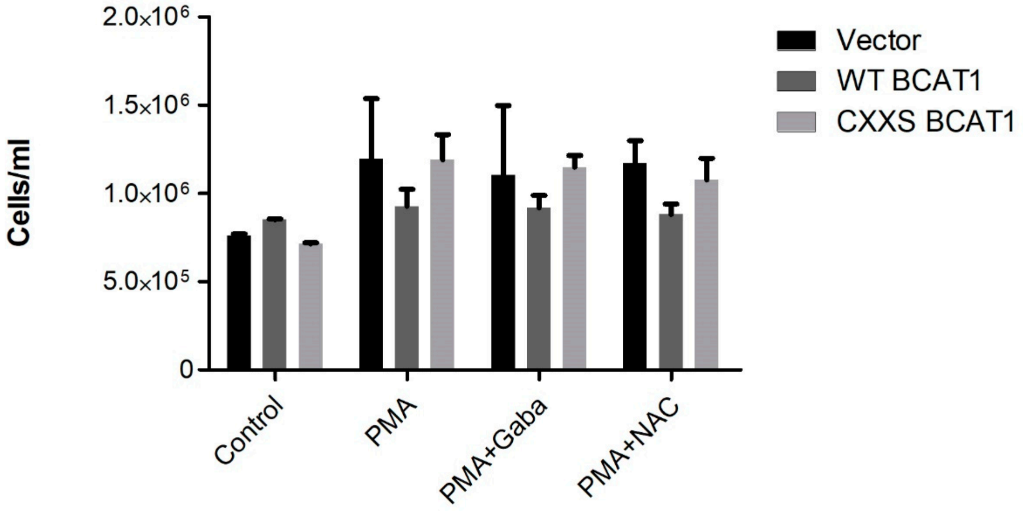

Supplemental Figure S5. The effect of PMA, Gabapentin (Gaba) and N-acetyl cysteine (NAC) on the viability and cell density of the retroviral transformed U937 cells a) Cells were incubated with 10nM PMA, 10nM PMA plus 20mM Gaba or 10nM PMA plus 10mM NAC in complete PPMI for 72 hours before viability was evaluated at point of immunophenotyping (see Figure 7). **b)** Data illustrates cell cells/ml following treatment

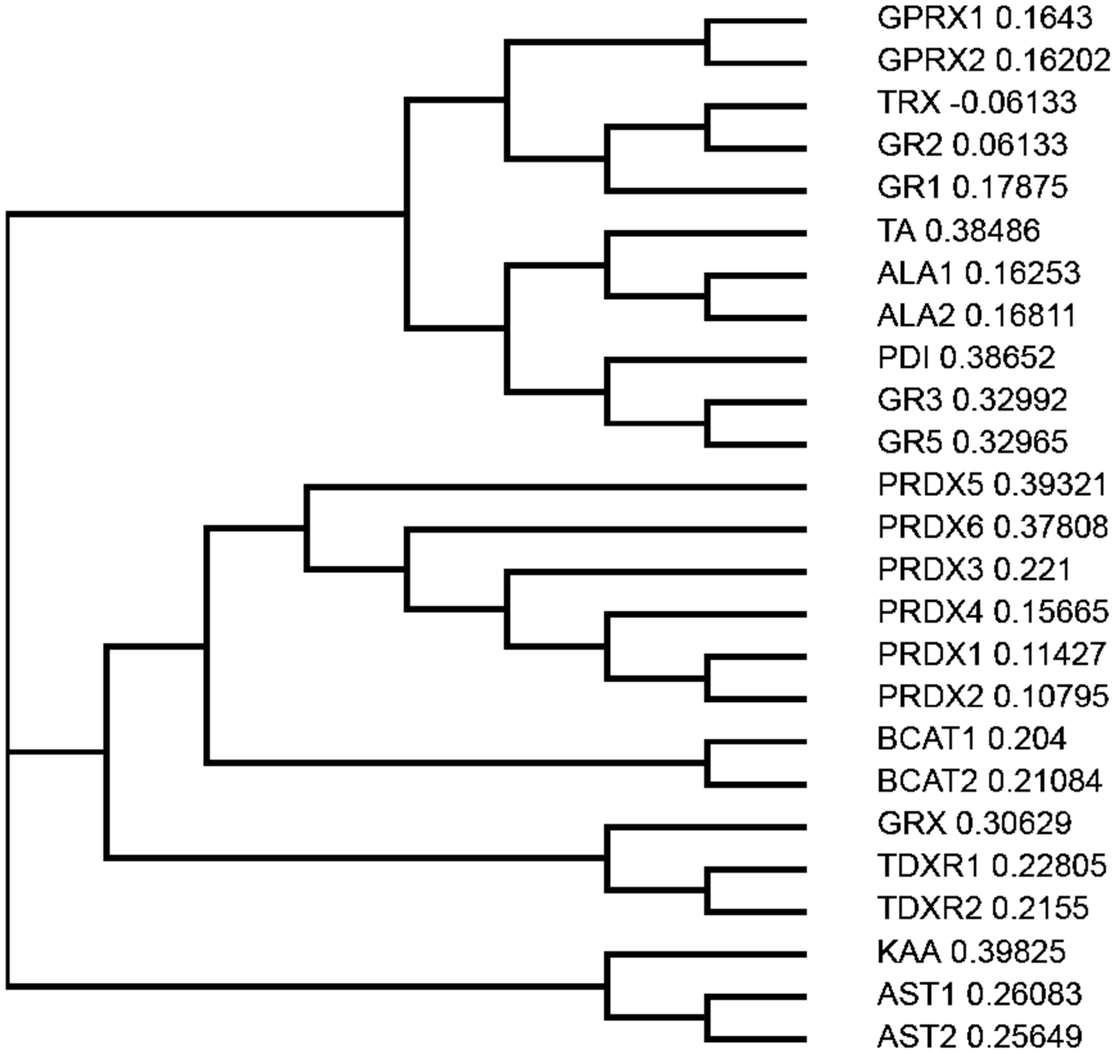

Supplemental Figure S6. Cladogram of human Cys antioxidant and human PLP dependent aminotransferase proteins. GPRX1 - Glutathione peroxidase 1, GPRX2 - Glutathione peroxidase 2, TRX - Thioredoxin 2, GR2 - Glutaredoxin 2, GR1 - Glutaredoxin 1, TA - Tyrosine aminotransferase, ALA1 - Alanine aminotransferase 1, ALA2 - Alanine aminotransferase 2, PDI - Protein disulphide isomerase, GR3 - Glutaredoxin 3, GR5 - Glutaredoxin 5, PRDX5 - Peroxiredoxin 5, PRDX6 - Peroxiredoxin 6, PRDX3 - Peroxiredoxin 3, PRDX4 - Peroxiredoxin 4, PRDX1 - Peroxiredoxin 1, PRDX2 - Peroxiredoxin 2, BCAT1 - Branched-chain aminotransferase (cytosolic), BCAT2 - Branched-chain aminotransferase (mitochondria), GRX - Glutathione reductase, TDXR1 - Thioredoxin reductase 1 (cytosolic), TDXR2 - Thioredoxin reductase 2 (mitochondrial), KAA - kynurenine/alpha-aminoadipate aminotransferase (mitochondrial), AST1 - Aspartate aminotransferase (cytosolic) and AST2 - Aspartate aminotransferase (mitochondria).

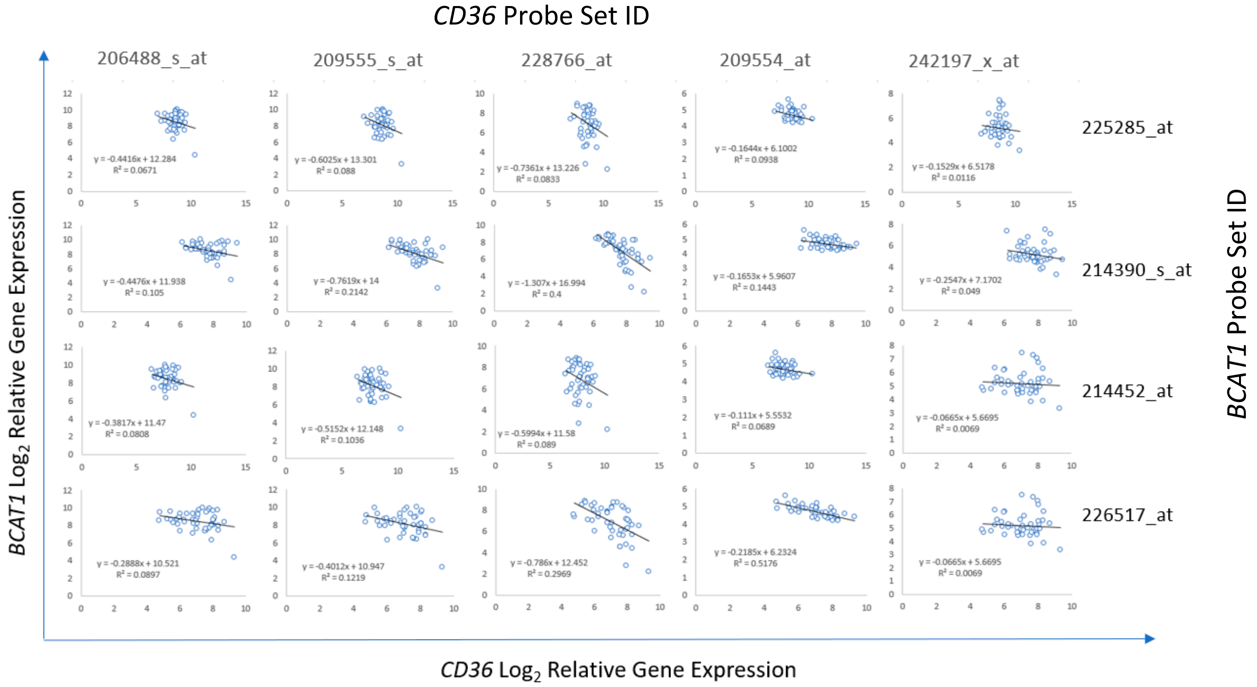

Supplemental Figure S7. Correlation between *BCAT1* and CD36 relative gene expression. Relative gene expression analysis was performed using BloodSpot data sets. The data presented are AML inv16 patients, which illustrate a consistent negative correlation between *BCAT1* gene expression level and *CD36* gene expression level, for all respective probes-set analysed.
